# Laboratory Experience in the Bacteriology of Diphtheria1Read before the Pathological Section of the Buffalo Academy of Medicine, December 17, 1895; appending circular recently published by the Health Department.

**Published:** 1896-03

**Authors:** William G. Bissell

**Affiliations:** Bacteriologist, Department of Health, Buffalo, N. Y.


### LABORATORY EXPERIENCE IN THE BACTERIOLOGY OF DIPHTHERIA.[1]

By WILLIAM G. BISSELL, M. D.,

Bacteriologist, Department of Health, Buffalo, N. Y.

A CONSIDERABLE amount of bacteriological work relating to cultural examinations for the diagnosis of diphtheria was carried on in the laboratory of the Department of Health in this city prior to January, 1895, but it was not until about that time that such work was begun in a regular and systematic manner.

The importance of such examinations has been thoroughly demonstrated by the uniform result of experiments carried on throughout the United States, England, Germany and France, and the time has arrived when the exact value of such examinations can be clearly and definitely stated.

The scope of the work carried on in the Bacteriological Bureau of the Department of Health in this city is familiar to most of the members of this section of the academy, and I will not impose upon your time by going into detail as to the system used, but will confine myself particularly to the points pertaining to the bacteriology of diphtheria, as experienced in making over 1,500 cultural examinations.

The object sought in routine municipal bacteriological examination for the diagnosis of diphtheria, is to promote general sanitation and thereby lessen the death rate. The individual points tending toward this ultimate result are as follows:

1. The confirming of clinical diagnoses and the determining of doubtful cases.

2. The differentiating between the cases of true diphtheria and those caused by cocci, so that persons affected by the latter complaints may not be unnecessarily exposed to diphtheria, as in the case of being sent to a hospital ward, or undergo the inconvenience of being isolated and their dwellings placarded, in case of their remaining at home.

3. To determine the presence or the absence of the specific bacillus in the secretion from the parts previously affected in convalescent cases, the object being to lessen the spread of the disease by not letting such persons at large until the infectious element has disappeared.

My paper this evening will present briefly an account of the

1. Read before the Pathological Section of the Buffalo Academy of Medicine, December 17, 1895; appending circular recently published by the Health Department.

work performed in this city by the Department of Health by summarising such facts as would seem likely to be of interest and of value pertaining to the bacteriology of diphtheria.

It might be well to first explain in a general way the various methods by which routine bacteriological examination is carried on. There are principally three:

1. By using a culture set, composed of a sterilised swab and the Loeffler blood serum media, the culture being made at the bedside of the patient by the attending physician or those in charge.

2. By supplying the practitioner with a sterilised swab made of lamb's wool. The exudate being collected on the swab and such swab, encased in a sterilised glass tube, sent directly to the laboratory. The bacteriologist makes an examination of the exudate direct before making a cultural inoculation and subsequently examining the cultural growth.

3. By using a platinum wire attached to a glass rod, commonly called an oese, the physician or attendant obtaining the infected material from the throat by the use of the oese instead of the swab, the inoculation being made at the bedside of the patient as in the first-named method.

The objections to the method of using the oese and the one of sending the exudate on the swab directly to the laboratory are apparent.

By an inexperienced person the use of the oese is a difficult and painstaking procedure, and cultural inoculations made in this manner by the ordinary practitioner are very apt to be inferior and more uncertain as to obtaining a good inoculation than those made by the use of the swab.

As to the method of sending the swab containing the exudate direct to the laboratory, it would be impossible in this way to carry on routine municipal examination as it is conducted in the city of Buffalo. The swab must be delivered direct to the laboratory in order that it may not become dried, and this is ofttimes inconvenient or impossible for physicians to do.

The point has been raised as to whether it is possible by microscopical examination of a smear of the exudate to make a reliable and positive diagnosis as to the existence of diphtheritic infection. Such examination would oftentimes be of the greatest value to the physician by the immediate determination of a suspicious case, but the procedure is so uncertain as to reliable results that I do not favor this method for routine work.

Oftentimes only a few organisms are present in the exudate at the time of its collection and it is an easy matter for an experienced observer not to detect their presence. So, considering the inconvenience of being obliged to send the swab directly to the laboratory, and the unreliability as to the result of the examination and the universally good results obtained by the use of the swab cultural inoculation, I am led to believe that the original method, as suggested by Dr. Biggs, of New York, is the most satisfactory in that it is most convenient and much more reliable.

Experience has demonstrated in the laboratory work in this city that persons who have once had experience in making cultural inoculations, and who follow carefully the points laid down in the directions for making such inoculations, invariably get satisfactory inoculations.

Out of 1,535 cultural inoculations made by the physicians in this city since the inauguration of municipal bacteriological examination, but eighty-three were found to be imperfect, and in about one-third of this number the swab had been thrust into the media, showing that the physician had not carefully read the directions, as such portion is noticeably printed in italics.

Occasionally a culture will be so highly contaminated with various bacterial growths that it is impossible to recognise the Klebs-Loeffler bacillus, when such organism is present in small numbers.

The smallest amount of certain germicidal fluids, especially the solutions of the bichloride of mercury, in the throat of a patient at the time of the collection of the exudate will frequently prevent growth on the culture media, and it is important to bear in mind this condition when obtaining material for cultural inoculation.

As to the best media to use in routine diphtheritic examination, I think it is the universal opinion of workers in this line that the Loeffler blood-serum, prepared by the quick method, answers every purpose. Much has been said relating to the value of the bouillonised hydrocele fluid as an isolating media in such examinations. It was claimed that the Klebs-Loeffler bacillus is of more rapid growth than the commoner cocci usually found in the throat of diphtheritic patients, and that such organism, when present in but small numbers in the exudate, will predominate over the other organisms on cultural examinations. I do not think this to be a fact, for carefully conducted experiments as to the isolating power of this media in relation to the Klebs-Loeffler bacillus tend to show that the commoner cocci develop about as rapidly as does the diphtheria organism.

The Klebs-Loeffler bacillus grows rapidly on various media used in bacteriological work, glycerine agar probably being the most suitably adapted next to the blood-serum mixture.

As to the descriptive characteristics of the diphtheria bacillus, I will not report at length, but I wish to emphasise the fact of the great liability to variations in its form and size. This tendency to change is sure to puzzle the inexperienced observer, though when understood becomes in itself microscopically diagnostic. The organism as found in the exudate ofttimes presents the appearance of a diplobacillus, which stains more or less uniformly, usually showing some polar darkening. In other and much rarer cases it resembles the organism as grown on blood-serum. The cause of this variation may possibly be found in the condition as to the reaction or composition of the mucous membrane or secretion in the throats of different patients. This idea seems very plausible, for under artificial cultivation the character of the media greatly influences the appearance of the bacillus; but the variations shown in the bacilli of cultures cannot be entirely accounted for by the character of the media, or by the temperature at which it was grown, or the staining manipulation, for the cultures of different exudates grown together under precisely similar conditions and stained in like manner often give different results. In some the bacilli may be uniform in size, shape, arrangement and staining, while others will exhibit wide differences in all these respects. The most typical form of the diphtheria bacillus is usually the one grown on blood-serum media, presenting extremely long, narrow rods with wide interruptions usually stained heavily at the ends and of irregular length.

I have noticed that in media containing much water of condensation the staining peculiarities are usually not as well marked, the most typical form of this bacillus being usually found in growths on media that is a week or so old and slightly dried. The best temperature for the rapid development of the Klebs-Loeffler bacillus seems to be that of the body or about 36½ centigrade, and it is usually necessary for cultures to remain from ten to sixteen hours at this temperature to obtain complete development.

The result obtained from the examination of 1,535 cultures shows that in Buffalo the greater majority of the cases of diphtheria yield cultures of the large size bacillus, which is usually characteristic in shape and manner of staining. It was at one time thought that the length of the organism had to do with its virulence, but experiments tend to show that this has little practical bearing. Out of the 1,535 cultures examined, 412 revealed the presence of the Klebs-Loeffler bacillus, 1,040 did not reveal the presence of the Klebs-Loeffler bacillus, and, as I have previously stated, 83 inoculations would not permit of examination.

Of the 1,040 cultures which did not reveal the Klebs-Loeffler bacillus, the organisms found, named in order of the frequency of their occurrence, are as follows :

No. 1.—Staphylococci, the most numerous being the aureus.

No. 2.—Cocci without any definite arrangement.

No. 3.—Streptococci.

No. 4.—Bacilli other than the Klebs-Loeffler (see note No. 2), and deserving of special mention ; a very large strepto-bacillus is of frequent occurrence.

No. 5.—The thrush fungus.

No. 6.—Diplococci.

The micrococci tetragenus has presented itself in several cultures, but such organism is not of frequent occurrence.

It is a noticeable fact that in several cases where the physician had noted on the report blank that a decided membrane was present in various portions of the mouth, that the auideum albicans would be present in the culture in goodly numbers.

Statistics reveal some very interesting facts concerning the influence of age on the occurrence of true diphtheria, as well as to the mortality of the disease. The ages of persons attacked between January 1, 1895, and December 1st, range between three weeks and about sixty years. The number of cases seem to increase with each twelve months of life up to the fifth year, and then gradually diminish. The mortality is highest in the first three years of life and then steadily diminishes until adult life. There have been two deaths this year of persons between the ages of fifty and sixty years in the city of Buffalo.

The ages and mortality in 728 cases are as follows :

| Month. | Deaths as to Ages. | | | Number of cases reported. | Number of cases discovered by bacteriological examination. |
|---|---|---|---|---|---|
| | Under 5. | 5 to 10. | Over 10. | | |
| January | 23 | 6 | 3 | 77 | 27 |
| February | 8 | 3 | 4 | 48 | 15 |
| March | 14 | 3 | — | 58 | 10 |
| April | 7 | 2 | 1 | 43 | 17 |
| May | 3 | 2 | 1 | 39 | 21 |
| June | 6 | 3 | 1 | 48 | 29 |
| July | 11 | 2 | — | 48 | 30 |
| August | 12 | 1 | 2 | 48 | 33 |
| September | 15 | 5 | — | 54 | 30 |
| October | 23 | 9 | 1 | 114 | 58 |
| November | 28 | 10 | 1 | 142 | 59 |
| | 150 | 46 | 14 | 728 | 329 |

Total number of deaths—210.

In cases that have died of diphtheria, in which bacteriological examination had been made at the onset of the malady, it may be a matter of interest and value to note that cultural examinations *usually* did not reveal pure cultures of the Klebs-Loeffler bacillus, there being associated with that organism either staphylococci or streptococci. Another point worthy of consideration and certainly of the greatest interest, is that in the majority of instances where the attending physician would report that the case was very mild and hardly in a condition to be called ill, that both the staphylococcus and the streptococcus would be associated with the typical Klebs-Loeffler bacillus. (See note No. 1.)

The point I wish to bring up for consideration is that the greatest mortality in cases of diphtheria appears to be produced by a mixed infection ; that is, the specific germ is usually associated with either the streptococcus or the staphylococcus, the former organism being of the greater frequency.

It seems also to be a fact that when both the staphylococcus and the streptococcus are associated in the same culture with the diphtheria bacillus, that the case is a mild one. From this latter point it would certainly appear as if there was a certain antagonism between the combined cocci and the diphtheria bacillus, and this latter point seems worthy of consideration and experiment.

As to the length of time the Klebs-Loeffler bacillus may remain alive in the throat of convalescent patients, experiments in the city's laboratory have demonstrated this time to be from about six days to thirty-nine days in the cases examined. There is no doubt, however, that the organism can remain a much greater length of time and be capable of producing the disease, and it is of the greatest importance, from the sanitary point of view, that bacteriological examinations should be made to determine the presence or absence of the infectious element before convalescent patients are allowed to go at large.

Many experienced physicians still find difficulty in believing that cases in which the exudate or pseudo-membrane is entirely absent from the pharynx and tonsils are those of true diphtheria, and it is also difficult to impress upon parents that a case is diphtheria and capable of transmitting the infection, although the person infected is hardly in a condition to be called ill.

In conclusion, I may state that absolute reliance can be placed on bacteriological examination, by a competent bacteriologist, of the bacterial growth on the blood-serum media, which has been properly inoculated and kept for about fourteen hours at the body temperature, in cases where there is a visible membrane in the throat, if the culture is made during the period in which this membrane is forming and no antiseptic, especially mercurial solutions, has been recently applied.

In cases where the membrane is confined to the larynx or bronchi, and where, therefore, there is no visible exudate against which the swab can be rubbed, surprisingly accurate results can be obtained from cultural examinations, but in a small proportion of such examinations the diphtheria bacillus may not be found in the first culture, whereas subsequent cultures will reveal them in goodly numbers. It is a fact, therefore, that negative results in laryngeal cases are not absolutely reliable. In nasal diphtheria, cultures made from the throat will frequently not show the bacillus, whereas those made from the exudate obtained from the nose will reveal them in large numbers. In making a diagnosis for diphtheria it is important to know the duration of the disease, because, although the bacillus may not be present in the culture examined, it may have been present in the throat at some former time in the course of the disease.

It is a rule, however, that the diphtheria bacillus will remain for some time in the throats of patients after the disappearance of all membrane and constitutional symptoms.

From the points previously stated it is necessary to know the location of the membrane, for negative results in laryngeal cases are not absolutely reliable.

There is yet to be a case examined in the department of health that has been reported as false diphtheria, which has died of the disease.

[NOTE No. 1.—On looking through literature pertaining to the bacteriology of diphtheria, I notice that other observers have noticed this same condition, such observers being both in this country and in Europe.—W. G. B.]

[NOTE No. 2.—During the discussion of this paper one member of the Academy expressed regret at the author not having referred to the "other organism which closely resembles the diphtheria bacillus—namely, the pseudo diphtheria bacillus." The failure to refer to this so-called "pseudo diphtheria bacillus" was intentional, for in the author's judgment positive proof as to the existence of this bacillus is still wanting. It seems most possible and probable that the referred-to organism is none other than the Klebs-Loeffler germ, altered in virulence and in certain other slight characteristics.—W. G. B.]

### DEPARTMENT OF HEALTH, BUFFALO, N. Y.

#### GENERAL RULES FOR THE PREVENTION AND RESTRICTION OF DIPHTHERIA.

Diphtheria, sometimes known as diphtheritic croup; membraneous croup; diphtheritic sore throat and croup, is one of the most dangerous and fatal diseases known to mankind.

It is very contagious, such contagion being transmitted principally in three ways, viz.:

(1). By personal contact with persons, and the attendants of patients, having the disease.

(2). By contact with the discharges (secretion and excretion) from the affected parts of persons having diphtheria.

(3). By articles of clothing, bedding, carpets, dishes, books, etc., which have been in the sick-room and liable to be infected.

The disease can be transmitted by articles of food, such as milk, bread, etc., and persons having diphtheria, or in attendance on such patients, should not be allowed to peddle or distribute such food, as it is a means of spreading the contagion.

*The Cause.*—The active agent in the production of the disease, is a germ or microbe, called the bacillus of diphtheria, and it is the germ alone that can cause true diphtheria.

The germ first fastens itself in the throat, nose or some other mucous membrane of the body, and as it grows produces a deadly poison which is absorbed by the system.

It is usually necessary to have some lowering in the normal healthy condition of a person, or an abrasion (sore) on some of the mucous membranes, to allow of the invasion (taking) of the disease, and this partially explains the reason why frequently one person will contract the disease, whereas another person equally exposed does not.

Children under the age of fifteen years are most susceptible to diphtheria, but adults are not exempt and they frequently contract the disease with fatal results.

*The Disease.*—Although diphtheria is a disease which usually makes the patient very sick, yet it does not always do so, and frequently persons having the disease are up and around and hardly in a condition to be called sick. It is these very mild cases that furnish the greatest source of danger as to spreading the contagion.

Such cases are as capable of transmitting the disease to others, providing the germ be present and alive in the throat, as more severe cases, and such person should remain in the house and be isolated until the germ has disappeared.

During the year 1894, 733 cases of diphtheria were reported to the Department of Health, and 186 of that number proved fatal.

One can appreciate, by this number, the mortality of the disease.

*How to Prevent the Disease from Spreading.*—Every case of diphtheria is dangerous to life. A physician should be called early and his directions carefully followed.

In case of the poor who are unable to pay for medical services, apply immediately to the Poor Department and a City Physician will be sent to attend the person free of charge.

The sign "DIPHTHERIA" placed upon a house is intended for the protection of the public and its warning should be heeded. More than half the cases of this sickness are contracted because of some one's carelessness, and if all persons interested would heed the warnings given them, its spread might be decidedly limited, for the disease is preventable.

Never enter a house having such sign unless absolutely necessary. Avoid coming in contact with the attendants of persons having the disease.

The patient must be kept in a room alone and no one admitted to that room except those in immediate attendance on the patient. It is well to quarantine the nurse with the patient and such nurse should not mingle with other persons of the household. No person, under any circumstances, should occupy the same bed with a diphtheritic patient. Doors communicating with the sick-room should be kept closed, and it is an excellent plan to hang over the door a sheet moistened with a disinfecting solution.

Sufficient ventilation can usually be obtained through an open window. The best place to isolate a diphtheritic patient is in the uppermost portion of the house. The room in which the sick person is confined should be scantily furnished, containing only such articles as are necessary for the well-being of the patient. All draperies, carpets, rugs, curtains, pictures, etc., should be removed and the bed clothing should be of the oldest, so that after the disappearance of the infection such clothes may be destroyed by fire. All closets and bureaus should be emptied before placing the patient in the room. The soiled clothing from the sick and the attendant should not be included in the family wash, and such clothes should be placed in a germicidal solution immediately after use and before going through the laundry.

Everything that the patient touches or comes in contact with is a source of danger to outsiders, and all secretions and excretions are especially dangerous. The chamber and bed-pan should be kept half full of a germicidal fluid, and all dishes, spitting cups, medicine bottles, etc., should be thoroughly disinfected before leaving the sick-room, and thoroughly washed in boiling water afterwards. Never use the same dishes on the family table that are in use in the sick-room. Children who have passed safely through an attack of diphtheria should be kept out of school until bacteriological examination fails to reveal the presence of the germ in the secretions from the affected parts. Such examination will be made *free of charge* by the Department of Health for the attending physician, who can obtain the material necessary for the examination at any police station. The germ of diphtheria remains in the throats of persons having had the disease for weeks and frequently months after recovery, and such persons are the greatest source of danger of infection to others. For a similar reason, the convalescent or recently recovered child should not be allowed to mingle freely with his playmates for the same period of time after recovery. It is important that during this time attendance at school should also be forbidden to all children who have lived in the same house with the patient. The school-room must be carefully watched, lest it become a place for the transmission and spreading of infectious disease.

The interments of persons who die of diphtheria must be private, and the corpse should not be exposed to view after it has been placed in the coffin ; moreover, it should be placed in the coffin and buried at the earliest possible moment. Every moment that a diphtheria corpse remains unburied is a menace to the lives and health of the survivors. The body should, immediately after death, be wrapped in a cloth saturated with a strong solution of corrosive sublimate and must be buried within twenty-four hours.

No one should ever, under any circumstances, kiss a body dead of diphtheria ; whoever does so is liable to contract the disease in a very severe form.

These precautions will do very much to prevent the spread of the disease among the survivors. It is a matter of record that carelessly conducted funerals have more than once been the starting point of a fatal epidemic of diphtheria among those who have attended such funerals.

### DISINFECTION.

This means the destruction of the disease germ, and consequently preventing the spread of the disease. The best way to disinfect articles is to destroy them by fire or submit them to the action of live steam. Those which it is desired to preserve are usually rendered harmless by thoroughly soaking for an hour in either of the following solutions:

1. Corrosive sublimate, one drachm; hydrochloric acid, one ounce; water, one gallon; or

2. Carbolic acid, six ounces; water, one gallon. Label each, "POISON."

A basin of ordinary soap and water and a basin of one of these solutions, half strength, should be constantly near for the nurse to wash her hands in. Any article infected by a patient should be placed in one of these solutions until it can be burned or thoroughly boiled in water.

In fumigating by burning sulphur, (using three pounds of sulphur to a room ten feet square, and increasing the amount according to the size of the room in proportions of three pounds to each additional 1,000 cubic feet of air space), the room should be tightly closed and allowed to remain so for at least twenty-four hours. The amount of sulphur burned is of great importance.

In proceeding to fumigate, the sulphur may be placed on a bed of cold ashes contained in an iron pot or coal scuttle; this is then put in a wash-tub holding an inch or two of water. The sulphur may be ignited with a shovel of glowing coals, or it may be lighted by a match after moistening its surface with alcohol. Take care not to breathe the sulphurous fumes. The sulphur acts more surely as a disinfectant on articles which are moistened with water, but it is then apt to destroy the color. Thoroughly air the room after fumigating with sulphur. The attempt to disinfect the air of a room while occupied by the sick person is absurd and useless, but the room should be kept ventilated by the entrance of air through an open window.

*Physicians are Required by Law* to immediately give notice of the first case and of every case of a "disease dangerous to the public health," to the Department of Health and the physician in charge of this disease should coöperate for its restriction.

The house should be placarded for protection of the public, and such means of isolation and disinfection as outlined above should be enforced, in order to prevent the spread of disease.

*Law Pertaining to the Abatement of Contagious Diseases.*—"No person suffering from any of the diseases named in Section 11, and no person in charge of such patient shall attend any public, private or Sunday school, or any public place, or enter any public conveyance. Nor shall any such person expose himself or herself in public streets, or n any manner aid in spreading their malady.

"Nor enter any public conveyance without first notifying owner, driver or person in charge, who shall provide for its disinfection before again permitting its use; but no hack or public conveyance shall permit the entrance of any one suffering from small-pox, typhus fever or any other pestilential diseases."

*Law Pertaining to the Reporting of Contagious Diseases.*—"It shall be the duty of any physician immediately upon the first visit to report to the Health Department all cases of infectious or contagious diseases.

"Any person acting as nurse or midwife in charge of infectious or contagious disease, in case the services of a physician are dispensed with, shall likewise report.

"Every proprietor or person in charge of a hotel, boarding-house or lodging-house shall report immediately upon the discovery of infectious, pestilentious or contagious diseases, and any person knowing of, or having reason to believe of the existence of a case of infectious or contagious disease not reported or concealed, shall report the same to the Department.

"An officer in charge of any prison, asylum, or public institution of any kind, shall report immediately any cases of infectious or contagious diseases arriving, and each additional case as discovered, and once a week a report of all cases, upon death or recovery, the date and details of the disposition of the case according to the rules of the Health Department."

*Law Pertaining to Children Attending School.*—"It shall be the duty of the principal to exclude from school all pupils coming from a house where a contagious disease exists, or in which a death had occurred from such cause. Said pupils shall not be readmitted until there has been a compliance with the rules of the Department of Health governing the admission of pupils to schools. When a principal has reason to suspect existence of any contagious disease in a house, he shall immediately suspend pupils living therein, until the case has been investigated and shall report the same to the Department of Health. Contagious diseases shall include diseases of the skin, eye, itch, mumps and whooping cough."

*Law Pertaining to Burial of Persons Dying of Contagious Diseases.*—"No undertaker shall use any vehicle other than a hearse for conveyance of the body of any person dying from any of the specified infectious or contagious diseases.

"Nor shall the body of a person dying of infectious or contagious diseases be carried into any church, hall or any public place.

"The body of no person dead from infectious or contagious diseases shall be brought into the city without special permit."

"In case of death from any pestilential disease, small-pox, scarlet fever, diphtheria, yellow fever, cholera, typhus, etc., it shall be the duty of the person in charge of such deceased to cause him or her to be buried within twenty-four (24) hours, and the person in charge of the funeral of persons dying of these diseases must so conduct such funeral as to be absolutely private."

*Law as to Placarding of Houses.*—"It shall be the duty of the Department of Health to put up and maintain in a conspicuous place in front of any building in which there shall be any person sick or infected with small-pox, varioloid, scarlet fever or diphtheria, a card or sign on which shall be written or printed, in English and German, the words designating the contagious disease with which the sick person is afflicted, and shall keep the same so posted during all the time that any person so infected shall remain in said building.

"And no person shall remove such sign or card placed on said premises, without permission of the Health Commissioner.

"In case of removal of such sign by accident or design, it shall be the duty of the occupant of said building to immediately notify the Department of Health."

ERNEST WENDE, M. D., *Health Commissioner*.

